# An Hourglass-Shaped Wireless and Passive Magnetoelastic Sensor with an Improved Frequency Sensitivity for Remote Strain Measurements

**DOI:** 10.3390/s20020359

**Published:** 2020-01-08

**Authors:** Limin Ren, Moyue Cong, Yisong Tan

**Affiliations:** School of Mechanical Engineering, Northeast Electric Power University, Jilin 132012, China; renlimin@neepu.edu.cn (L.R.); 2201800312@neepu.edu.cn (M.C.)

**Keywords:** hourglass shape, wireless, passive, magnetoelastic sensor, strain measurements

## Abstract

The conventional magnetoelastic resonant sensor suffers from a low detecting sensitivity problem. In this study, an hourglass-shaped magnetoelastic resonant sensor was proposed, analyzed, fabricated, and tested. The hourglass-shaped magnetoelastic resonant sensor was composed of an hourglass and a narrow ribbon in the middle. The hourglass and the narrow ribbon increased the detection sensitivity by reducing the connecting stress. The resonant frequency of the sensor was investigated by the finite element method. The proposed sensor was fabricated and experiments were carried out. The tested resonance frequency agreed well with the simulated one. The maximum trust sensitivity of the proposed sensor was 37,100 Hz/strain. The power supply and signal transmission of the proposed sensor were fulfilled via magnetic field in a wireless and passive way due to the magnetostrictive effect. Parametric studies were carried out to investigate the influence of the hourglass shape on the resonant frequency and the output voltage. The hourglass-shaped magnetoelastic resonant sensor shows advantages of high sensitivity, a simple structure, easy fabrication, passiveness, remoteness, and low cost.

## 1. Introduction

The magnetoelastic material has been widely used in the field of sensors since its emergence. These sensors are based on different effects of the magnetoelastic material. These effects include the giant magnetoimpedance (GMI) effect [1,2], stressimpedance (SI) effect [3,4], the small magnetization rotaion (SAMR) technique [5], and the resonant magnetoelastic effect. A new tensductor sensor [6] was developed based on the magnetoelastic material in 2018. Among of these new sensors, resonant magnetoelastic sensors show advantages in chemical and force detecting fields due to its remoteness and passiveness characteristics [7].

Frequency sensitivity is a key parameter of resonant magnetoelastic sensors [8,9,10,11,12,13]. A high frequency sensitivity means that only few samples are needed in the test process [7,14,15,16,17]. The testing accuracy and resolution can be improved at the same time [18,19]. Therefore, lots of studies have focused on this research area.

Grime C.A. et al took advantage of a magnetoelastic material to detect the atmospheric pressure [20]. The magnetoelastic material was tailored into a rectangle shape and the frequency sensitivity was not studied in the research. Andrew DeRouin et al. pasted the magnetoelastic material onto a substrate to detect strain [21]. The magnetoelastic material was also tailored into a rectangle shape and the frequency sensitivity was not studied either. Steven Trierweile et al. altered the shape of the magnetoelastic material into rectangle arrays by using etching techniques [22]. The frequency sensitivity was boosted and the detecting scope was broadened. However, there was interference between neighboring magnetoelastic ribbons, and the method was only suitable for biological field detection. Venkatram Pepakayala changed the shape of the magnetoelastic material into a spring [23]. The frequency sensitivity of the sensor increased to 12.5 × 10^3^ ppm/mstrain, which was much higher than before. However, the micro-electro-mechanical system (MEMS) techniques need a special apparatus and the fabrication cost is very high. Scott R Green managed the magnetoelastic material by using a microprecision spark [24]. It needed a long fabrication period for complicated shapes.

Based on the analyses listed above, one conclusion that can be drawn is that there is not a low cost and easy fabrication method which can improve the frequency sensitivity effectively of the resonant magnetoelastic sensors. Therefore, an hourglass-shaped wireless and passive magnetoelastic sensor (HSMS) was proposed in this paper. Its frequency sensitivity was tested. In contrast to previous works, the HSMS was shown to have high sensitivity. Additionally, it was easy to fabricate using an ordinary measure and it had a low cost.

## 2. Materials and Methods

### 2.1. Materials

The magnetoelastic material used in the HSMS was Metglas 2826 MB, which contained 40% Fe, 38% Ni, 4% Mo, and 18% B (Fe40Ni38Mo4B18). The 2826 MB is a kind of amorphous metallic glass due to its disordered atomic-scale structure. The thickness of 2826 MB is only 28 µm which gives it superiority in resonance applications. The 2826 MB has a magnetoelastic coupling factor of 0.98 and a magnetoelastic coefficient of 11.7 ppm. The magnetic and physical properties of Metglas 2826MB are presented in Table 1.

### 2.2. Structure of the Proposed HSMS

**Figure 1:**
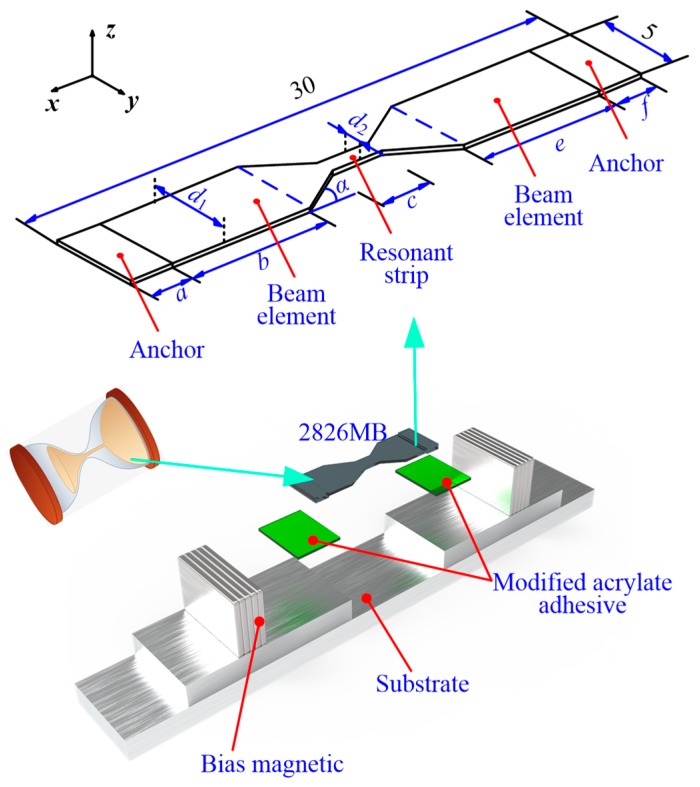
Structure of the hourglass shaped wireless and passive magnetoelastic sensor (HSMS).

Figure 1 shows the structure of the proposed HSMS. The HSMS is mainly composed of a sensing component 2826 MB and a substrate.

The material of the sensing component is magnetoelastic material. The sensing component was tailored into an hourglass shape using a Computer Numerical Control (CNC) milling machine (model: FF500-CNC, Föhren, Germany). The hourglass-shaped sensing component had a length of 30 mm and a width of 5 mm. There were two anchors on each end of the sensing component. Then, the sensing component was pasted on the substrate through the two anchors. The material of the substrate was aluminum alloy (Model: 7075, Shenzhen, China). The glue used in HSMS was modified acrylate adhesive (model: AILIKE-A8, Shenzhen, China). Two pieces of rare earth magnets were placed around the sensing component to provide a bias magnetic field [25].

**Figure 2:**
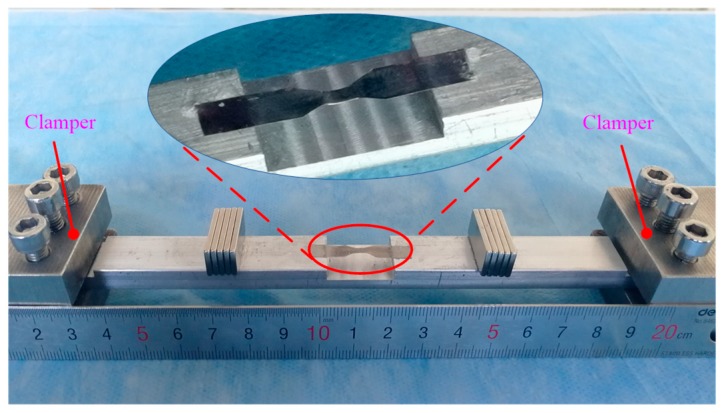
Prototype of the proposed HSMS.

The exact dimensions of the HSMS are given in Table 2. The dimensional and magnetic properties of the bias magnetic are shown in Table 3. A prototype was fabricated to verify the frequency performance of the HSMS, as shown in Figure 2. Two clampers were designed to fix the HSMS on both ends.

### 2.3. Finite Element Method (FEM)

**Figure 3:**
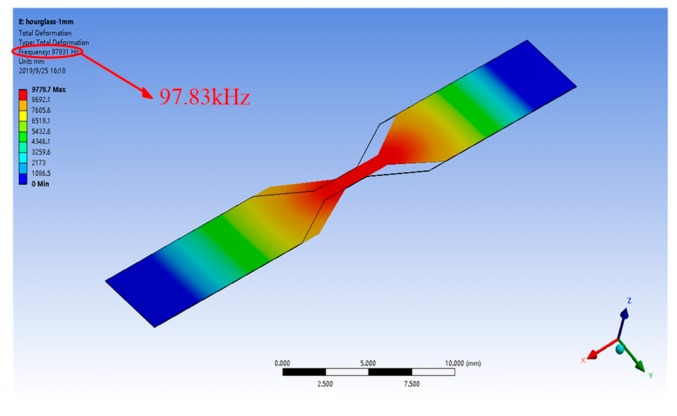
Resonant frequency determined by the finite element method (FEM) analysis.

A FEM analysis was carried out to acquire the resonant frequency of the HSMS. The mass density and Poisson’s ratio of 2826 MB were 7900 kg/m^3^ and 0.33, respectively. The resonant frequency provided by the FEM analysis is shown in Figure 3. The calculated frequency was 97.83 kHz. From Figure 3, it can be seen that the main vibration of the HSMS focused on the hourglass-shaped part which was shown by red color. That is also to say the shape of the hourglass is one factor that influence the frequency of the HSMS.

### 2.4. The Experiment Setup

**Figure 4:**
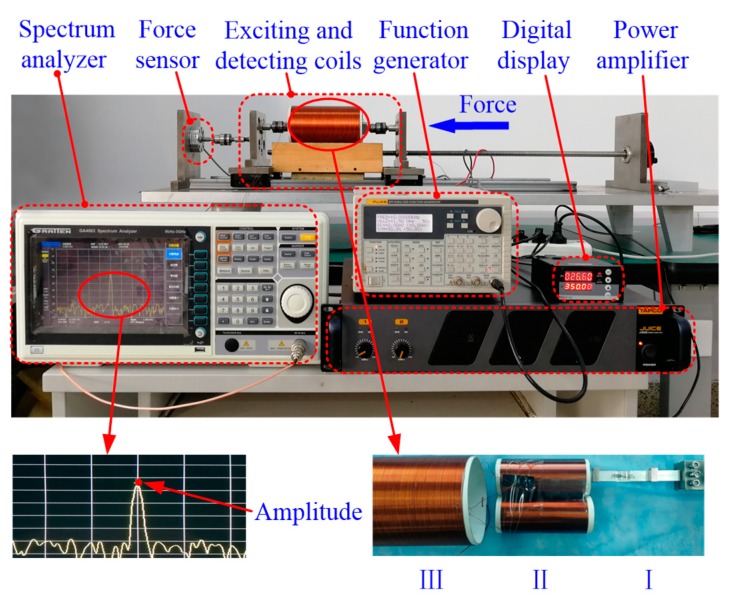
The experimental setup.

An experimental platform was designed and fabricated to obtain the frequency performance of the HSMS, as shown in Figure 4. A function generator (model: FLUKE-271, Washington, WA, USA) was used to generate a sinusoidal signal. The sinusoidal signal was amplified by a power amplifier (model: J.2500, New York, NY, USA). The amplified sinusoidal signal was input into an exciting coil to provide a working magnetic field. A detecting coil was placed around the HSMS to pick up the resonant frequency. The HSMS, the detecting coil, and the exciting coil are presented as I, II, and III in Figure 4. The strain was applied at the right by a handwheel and tested by a high precision force sensor (model: ZNLBM-500KG, Bengbu, China). The electric and dimension parameters of the exciting coil were 1 mm copper wire, 200 turns, and 110 mm external diameter. The electric and dimension parameters of the detecting coil were 0.4 mm copper wire, 200 turns, and 50 mm external diameter. Two detecting coils were connected reversely to eliminate the basic induction voltage from the exciting coil.

## 3. Results and Discussion

**Figure 5:**
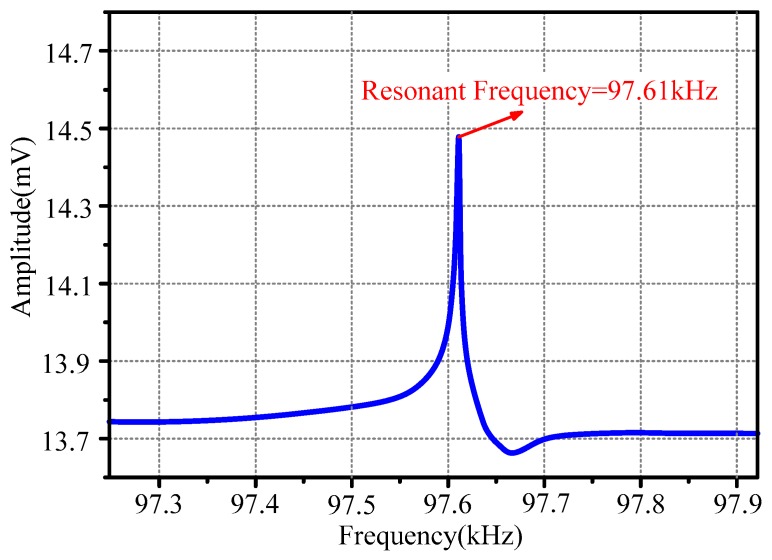
Actual resonant frequency of the HSMS.

Figure 5 expresses the actual resonant frequency of the HSMS. The actual resonant frequency was 97.61 kHz, and it was very close to the simulated one. The actual resonant frequency agreed with the simulated one which proved the design of the HSMS. There was a difference of 0.22 kHz, which was mainly caused by the glue used in the HSMS fabrication.

**Figure 6:**
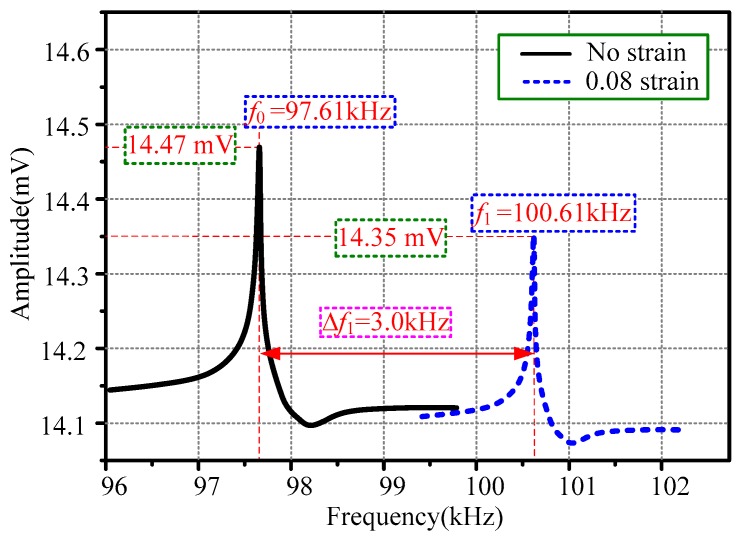
Frequency performance of the HSMS under a specific strain.

Figure 6 gives the frequency performance of the HSMS under a specific strain. The strain can be defined as follows:

(1) $$\varepsilon =\frac{\Delta l}{l}$$

where *ε* is the strain applied on the HSMS, *l* is the original length of sensing element, and Δ*l* is the varied length of the HSMS.

The solid black line shows that no strain was applied on the HSMS. The non-loaded resonant frequency was 97.61 kHz. The voltage obtained by the detecting coil was 14.47 mV. When a 0.08 tensile strain was applied on the HSMS, the resonant frequency became 100.61 kHz. It was represented by the dotted blue line. The voltage acquired by the detecting coil was 14.35 mV. The frequency shift of the HSMS under the tensile strain of 0.08 mm/m was calculated by

(2) $$\Delta f_{1}=f_{1}-f_{0}=100.61-97.61=3.0\;\mathrm{kHz}$$

where *f*_0_ is the resonant frequency under a strain of 0 mm/m and *f*_1_ is the resonant frequency under a strain of 0.08 mm/m.

The frequency sensitivity *δ* can be obtained by

(3) $$\delta =\frac{\Delta f_{1}}{\varepsilon }=\frac{3}{0.08}=37.5\;\mathrm{kHz}/\mathrm{strain}$$

The tensile strain applied on the HSMS can be seen as a mechanical preload which would alter the vibration status of the HSMS. The 3.0 kHz measured shift in the resonant frequency evidences that the HSMS could reflect the applied strain effectively. The voltage in the detecting coil changed from 14.47 to 14.35 mV. The voltage deviation of 0.12 mV was caused by the tensile strain.

**Figure 7:**
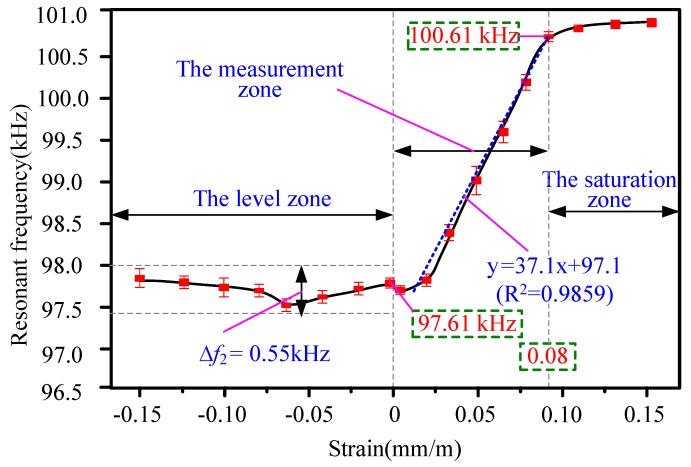
Plot of the resonant frequency of the HSMS versus the applied external strain.

Figure 7 is a plot of the resonant frequency of the HSMS versus applied external strain. The whole figure can be divided into three zones: the level zone, the measurement zone, and the saturation zone. The three regions indicate three different working statuses of the HSMS. In the measurement zone, the strain varies from 0 mm/m to 0.08 mm/m. The resonant frequency moves from 97.61 to 100.61 kHz. There is a linear incremental relationship between the resonant frequency and the applied external strain. The fitted curve is y = 37.1x + 97.1 by least-squares (R^2^ = 0.9859). The slope, 37.1 (unit: $\frac{\mathrm{kHz}}{\left(\mathrm{mm}/m\right)}$), is also the frequency sensitivity of the HSMS. Therefore, the strain region from 0 to 0.08 mm/m was selected as the measurement region due to its high linearity and high frequency sensitivity.

The saturation zone was a special region of the HSMS. Above 0.10 mm/m the resonant frequency did not increase significantly. This corresponds to the saturation zone of the HSMS according to the Metglas 2826 MB datasheet [26]. When the strain was over 0.15, the 2826 MB ribbon did not vibrate anymore, as it was totally saturated. Therefore, the saturation zone was not suitable for detecting purposes.

The level region in the figure was very different from the measurement and saturation regions discussed above. In the level region, a compressive strain was applied varying between 0 and 0.15 mm/m. From the figure, it can be seen that the resonant frequency did not change very much. The highest frequency shift was calculated by

(4) $$\Delta f_{2}=f_{h}-f_{l}=98-97.45=0.55\;\mathrm{kHz}$$

where $f_{h}$ is largest resonant frequency in the level zone, and $f_{l}$ is the smallest resonant frequency in the level zone.

Therefore, the frequency shift was very small in the level zone. Therefore, the HSMS was not suitable for the detection of compressive strain. The discussion above agrees well with previous studies [25].

**Figure 8:**
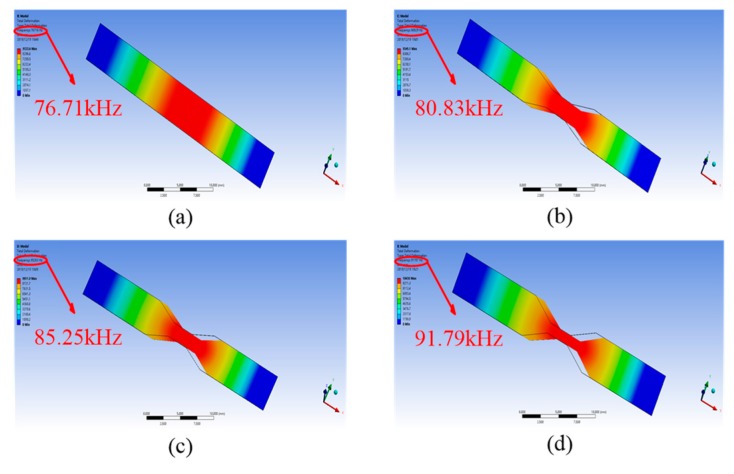
FEM analysis results of resonant frequency under different *d*_1_/*d*_2_. (**a**) *d*_1_/*d*_2_ = 1, (**b**) *d*_1_/*d*_2_ = 2, (**c**) *d*_1_/*d*_2_ = 3, and (**d**) *d*_1_/*d*_2_ = 4.

**Figure 9:**
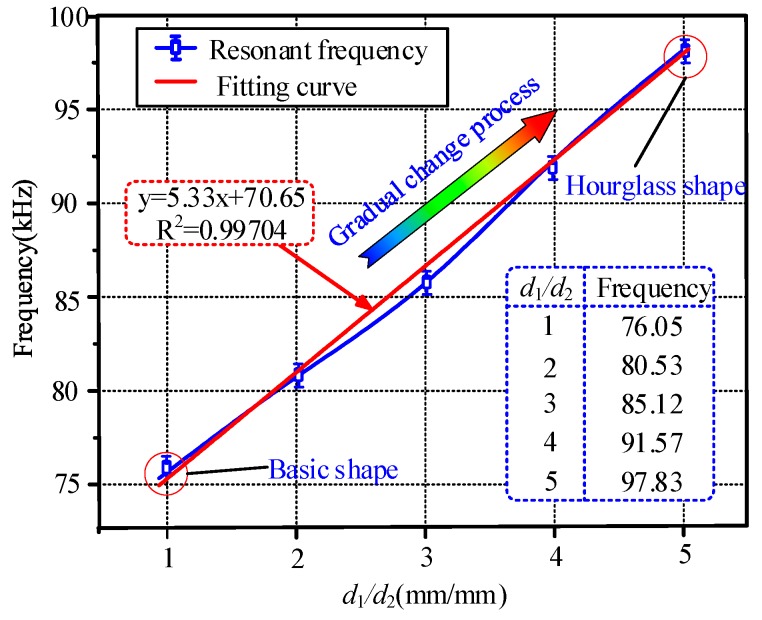
Plot of the no-load resonant frequency versus the ratio (*d*_1_/*d*_2_).

The influence of the hourglass shape on the resonant frequency of the HSMS was also investigated. The parameter *d*_1_ was set to 5 mm, and *d*_2_ varied from 1 mm to 5 mm. The FEM analysis results are given in Figure 8. The relationship between the no-load resonant frequency and the parameter ratio (*d*_1_/*d*_2_) is depicted in Figure 9. The overall relationship between the resonant frequency and the ratio was almost an incremental function. When *d*_1_/*d*_2_ was equal to 1, the resonant frequency was 76.05 kHz at this time, which agreed with the studies in [27,28] When the ratio was 2, the resonant frequency was 80.53 kHz, which was higher than that occurred at the ratio of 1. When the ratios were 3, 4, and 5, the resonant frequencies were 85.12 kHz, 91.57 kHz, and 97.83 kHz respectively. A linear fitting curve between the resonant frequency and the ratio (*d*_1_/*d*_2_) was obtained. The fitting curve was y = 5.33x + 70.65 by least squares. Figure 9 indicates further that the hourglass shape can heighten the resonant frequency of the HSMS.

**Figure 10:**
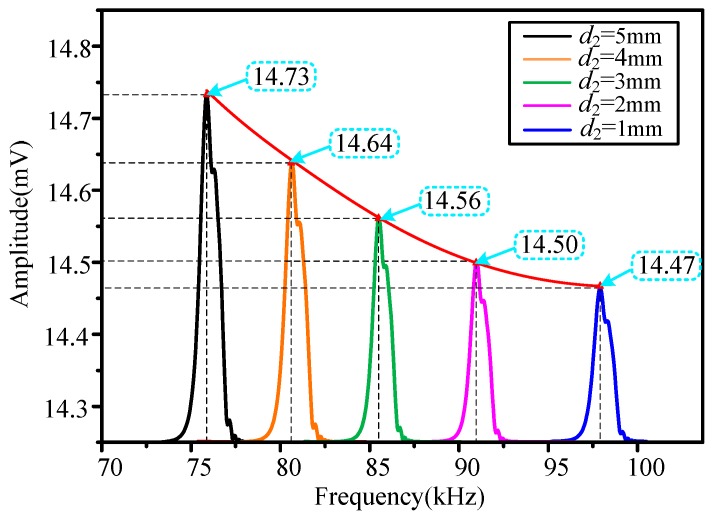
Plot of the voltage output versus the resonant frequency.

The voltage output at corresponding resonant frequencies was studied in Figure 10. The resonant frequencies were 76.05 kHz, 80.53 kHz, 85.12 kHz, 91.57 kHz, and 97.83 kHz respectively. The voltage outputs collected by the coil were 14.73 mV, 14.64 mV, 14.56 mV, 14.50 mV, and 14.47 mV. As the resonant frequency increased, the collected voltage decreased. A lower voltage output was disadvantageous to the signal-to-noise ratio of the HSMS. Therefore, it should be noted the simultaneous appearance of the higher resonant frequency and the lower voltage output puts forward a technical conflict. Our next research focus is to enhance the frequency sensitivity further and increase the voltage output at the same time.

**Figure 11:**
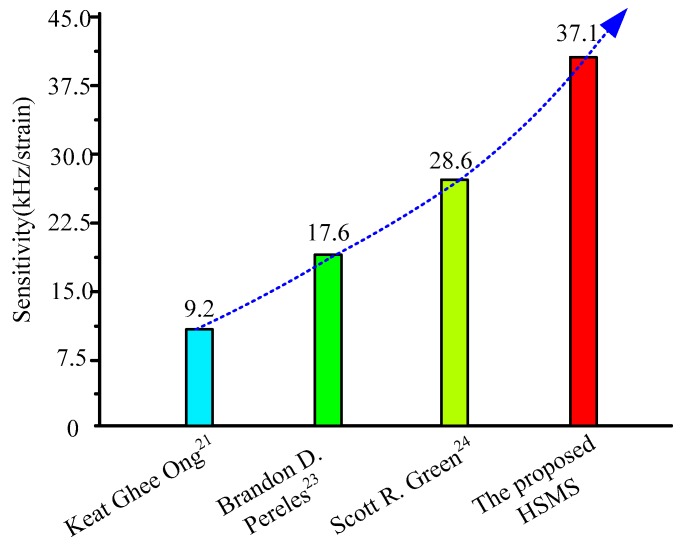
Frequency sensitivity comparison between the proposed HSMS and several other works.

Figure 11 shows the frequency sensitivity comparison between the proposed HSMS and several other works. The values of the frequency sensitivity of one unit strain in studies [21,23,24] were 9.2 kHz, 17.6 kHz, and 28.6 kHz respectively. The frequency sensitivity of the propose HSMS was 37.1 kHz. From the comparison, it can be concluded that the frequency sensitivity of the proposed HSMS is superior to the other three strain sensors.

## 4. Conclusions

In conclusion, this paper has described an hourglass-shaped wireless and passive magnetoelastic sensor with an improved frequency sensitivity. Compared to previous related works, the proposed HSMS had a frequency sensitivity of up to 37.1 kHz/strain. At the same time, the HSMS was easily fabricated using an ordinary CNC machine. The frequency performance of the proposed HSMS showed a linear incremental region which could be used as the measurement region. The HSMS with a large *d*_1_/*d*_2_ had a high frequency sensitivity. The HSMS could be applied for remote strain measurement due to its high frequency sensitivity.

## Figures and Tables

**Table 1:** Magnetic and physical properties of 2826 MB.

Magnetic Properties		Physical Properties	
Saturation Induction (T)	0.88	Density (g/cm^3^)	7.90
Maximum D.C. Permeability (µ):		Vicker’s Hardness (50 g load)	740
Annealed	800,000	Elastic Modulus (GPa)	100–110
As Cast	>50,000	Tensile Strength (GPa)	1–2
Saturation Magnetostriction (ppm)	11.7	Lamination Factor (%)	>75
Electrical Resistivity (µΩ·cm)s	138	Continuous Service Temperature (°C)	125
Curie Temperature (°C)	353	Thermal Expansion (ppm/°C)	11.7
Magnetoelastic Coupling Factor	0.98	Crystallization Temperature (°C)	410

**Table 2:** Dimensions of the HSMS.

Symbol	Parameter	Symbol	Parameter
*a* (mm)	2.5	*d*_2_ (mm)	1
*b* (mm)	8	*e* (mm)	8
*c* (mm)	3	*f* (mm)	2.5
*d*_1_ (mm)	5	*α* (°)	33.69

**Table 3:** Dimensional and magnetic properties of the bias magnetic.

Item	Properties
Length (mm)	20
Width (mm)	10
Height (mm)	2
Mass (g)	2.5
Magnetic Field Strength (T)	0.06
Material	Nd-Fe-B Rare Earth
